# Three-dimensional CBCT analysis of bone remodeling, root resorption, and incisive canal morphology during miniscrew-assisted aligner-based incisor retraction with and without extractions

**DOI:** 10.3389/froh.2026.1728205

**Published:** 2026-02-02

**Authors:** Remsh Khaled Al-Rokhami, Zhihua Li, Deguo Gao, Xin Zhao, Jingke Liu, Hua Fan, Karim Ahmed Sakran

**Affiliations:** 1 The Affiliated Stomatological Hospital of Jiangxi Medical College, Nanchang University, Nanchang, Jiangxi, China; 2 Zhenjiang Stomatological Hospital, Zhenjiang, Jiangsu, China

**Keywords:** bone remodeling, CBCT, clear aligners, incisive canal, maxillary central incisors, root resorption

## Abstract

**Objectives:**

This study aimed to investigate three-dimensional changes in incisive canal (IC) morphology, root–canal proximity, and root resorption following maxillary incisor (U1) retraction with clear aligners, comparing extraction and non-extraction treatment protocols.

**Materials and methods:**

A total of 100 patients (200 U1) treated with clear aligners were retrospectively analyzed using CBCT before and after treatment. The extraction group included 40 patients and the non-extraction group 60. Linear measurements of IC width, root–canal distance, cortical bone width (CBW), IC height, and root length/width were obtained at three levels above the labial cementoenamel junction (H1–H3: 2, 4, and 6 mm). Volumetric and surface area analyses of the IC and U1 were performed to evaluate morphological and structural remodeling. Group differences and predictive factors for root resorption and canal contact/invasion were assessed statistically.

**Results:**

Extraction-based retraction produced significantly greater reductions in canal width, CBW, and root–canal distance, with increased apical root resorption, most notably in patients who underwent substantial anterior retraction (all *P* < 0.05). The mean decreases in root–canal distance were 1.08 ± 0.92 mm, 1.10 ± 0.91 mm, and 1.02 ± 0.99 mm at H1, H2, and H3, respectively, in extraction cases, vs. 0.40 ± 0.58 mm, 0.47 ± 0.78 mm, and 0.30 ± 0.98 mm in non-extraction cases. Apical root resorption averaged 0.91 ± 0.80 mm in the extraction group and 0.32 ± 0.56 mm in the non-extraction group (*P* < 0.05), correlating with closer root–canal proximity. Volumetric and surface area analyses revealed greater reductions in IC and U1 dimensions in extraction cases, indicating intensified bone remodeling. Treatment duration, incisor movement, IC height, and root–canal distance were significant predictors of canal contact, while root resorption correlated positively with treatment duration, incisor movement, and root length.

**Conclusion:**

Extraction-based aligner retraction elicits greater osseous remodeling around the incisive canal and increased root resorption, particularly in cases requiring substantial anterior retraction, reflecting a coupled bone–tooth adaptive response to orthodontic loading. These findings highlight the dynamic nature of craniofacial bone physiology and emphasize the need for biologically guided treatment planning to minimize tissue stress and iatrogenic effects.

## Introduction

The maxillary central incisors (U1) play an essential role in facial esthetics, speech, and functional occlusion (1–3). Orthodontic retraction of these teeth must account for the anatomical constraints of surrounding structures. Although the anatomy of the incisive canal (IC) is well documented, its response to mechanical loading and its remodeling behavior during orthodontic movement have only recently been explored using three-dimensional imaging modalities. The canal, which houses the nasopalatine nerve and a vascular network, is surrounded by dense cortical bone and located in the anterior maxilla between the roots of the central incisors (4, 5).

Studies have shown that excessive tooth retraction may lead to root proximity or invasion into the canal, increasing the risk of root resorption and compromising long-term dental health (6–8). Assessing the incisive canal is therefore clinically essential, as encroachment or contact between the incisor roots and the canal may lead not only to root resorption but also to nasopalatine nerve irritation, reduced pulp vitality, and compromised stability of the orthodontic outcome. In one report, 53% of cases with >4 mm of retraction showed canal invasion, with an average root resorption of 2.39 mm and some cases exceeding 6 mm (9). Recent randomized controlled trials have further demonstrated that root resorption is strongly influenced by different anchorage systems and force vectors, with intrusive or skeletally anchored force systems producing significantly greater volumetric resorption in maxillary incisors (10). From a physiological perspective, such interactions reflect localized bone remodeling processes, where sustained orthodontic forces alter the balance between osteoclastic and osteoblastic activity within the canal walls and adjacent alveolar bone.

Clear aligner therapy offers aesthetic and biomechanical advantages but raises questions regarding its capacity to control root movement, particularly in extraction cases. Compared with fixed appliances, aligners may generate different magnitudes and distributions of mechanical stress, potentially influencing the pattern and extent of bone and root remodeling. Finite-element simulations expand this understanding, showing that with aligners, anchorage design, whether moderate, direct strong, or indirect strong, alter tooth stress distribution and posterior anchorage stability, suggesting that aligner-based retraction requires tailored biomechanical compensation to control root angulation during space closure (11). While previous studies using fixed appliances have highlighted canal-related root resorption, there is limited evidence describing the mechanobiological response of the incisive canal and surrounding bone during aligner-based retraction.

Studies using fixed orthodontic appliances have reported that excessive retraction of roots can cause the incisor roots to approximate or invade the canal, resulting in varying degrees of external apical root resorption and deformation of the canal cortices (6, 8, 9, 12). Previous CBCT investigations have shown reductions in canal width, thinning of the palatal cortical plate, and increased root–canal proximity in patients undergoing significant en-masse retraction with fixed appliances (8, 12). Other studies have demonstrated that when root–canal contact occurs, the risk and severity of apical resorption increase significantly compared with cases where sufficient clearance is maintained (6, 9). The influence of anchorage type on anterior tooth movement has been also validated in earlier clinical studies, where mini-implants provided significantly better molar stability and greater incisor intrusion compared with conventional anchorage during en-masse retraction (13).

The primary objective of this study is to assess three-dimensional changes in incisive canal morphology, root proximity, and resorption following clear aligner retraction in extraction and non-extraction protocols. A secondary aim is to identify factors influencing root–canal interactions, with particular attention to treatment-induced bone adaptation and root resorptive responses as indicators of craniofacial bone physiology under orthodontic loading.

## Materials and methods

### Study design and participants' selection

This retrospective study was approved by the institutional review board and adhered to the principles of the Declaration of Helsinki. Informed consent has been obtained from the patients at scanning time. Patient records were reviewed from a Stomatology Hospital for patients who had undergone maxillary incisor retraction using Invisalign® (Align Technology, California, USA) between January 2015 and January 2022.

The sample size was calculated using G*Power software, assuming a standard deviation of 0.85 mm and a mean difference of 0.8 mm in root-canal distance between the non-extraction and extraction groups (*α* = 0.05, power = 95%) (8), resulting in a required sample of 26 subjects per group. To improve reliability, a larger sample was included, totaling 100 consecutive patients. Patients were divided into extraction (*n* = 40) and non-extraction (*n* = 60) groups based on premolar extraction status. Specifically, the non-extraction group included patients with almost ≤2 mm horizontal maxillary incisor retraction, whereas the extraction group included patients with >2 mm incisor retraction following the removal of four first premolars.

Patients were included if they had dental/skeletal Class I (ANB 0 °–4 °) or Class II (ANB >4 °–8 °) malocclusions, high-quality pre-treatment (T1) and post-treatment (T2) CBCTs, and no prior orthodontic treatment. Exclusion criteria were: facial asymmetry (Menton deviation >2 mm), midline shifts >2 mm, maxillary midline diastema >2 mm, respiratory issues, pre-treatment root resorption or periodontal disease, missing teeth (except third molars), anomalies, trauma or surgery in maxillary midline, corticotomy, systemic disease, or medications affecting bone metabolism. To identify clinical profiles of enrolled cases, the American Board of Orthodontics (ABO) discrepancy index (DI) was used to evaluate case difficulty (14).

Incisor retraction mechanics were designed according to established biomechanical principles for clear aligner therapy. Miniscrew-assisted anchorage was used to approximate the line of action of force toward the estimated center of resistance (CR) of the anterior segment. TADs were placed between the maxillary second premolars and first molars, and elastic traction was applied from the TADs to precision-cut slots or bonded composite hooks on the aligners, thereby generating a more bodily retraction force and minimizing uncontrolled tipping (Supplementary Figure S1). TADs were used in all extraction cases and in non-extraction cases where ≥2 mm of planned incisor root control or bodily movement was required. Invisalign® with G6-optimized attachments was used, and patients wore aligners 22 h per day with six-week follow-up intervals. The staging protocol involved initial canine distalization followed by incisor retraction to optimize space management, enhance aligner force expression, and improve predictability of root control during anterior retraction.

All included cases achieved clinically acceptable alignment, incisor inclination, and midline control at T2, consistent with the planned digital setup, ensuring the clinical validity of the CBCT-based comparisons.

### CBCT acquisition and analysis

CBCT scans were acquired using standardized protocols (120 kVp, 5 mAs, 0.3 mm voxel size, 16 × 13 cm field of view) with the I-CAT Imaging System (Imaging Sciences International Inc., Hatfield, PA, USA). Scans were oriented to the Frankfort plane. All CBCTs were for diagnostic and evaluation purposes (e.g., root position, skeletal discrepancy, root integrity, teeth impaction, TMJ), mainly in cases requiring significant retraction, with no additional radiation for research purposes. T2 scans were superimposed onto T1 to ensure measurement accuracy and consistency of reference planes between pre-treatment and post-treatment scans.

**Figure 1:**
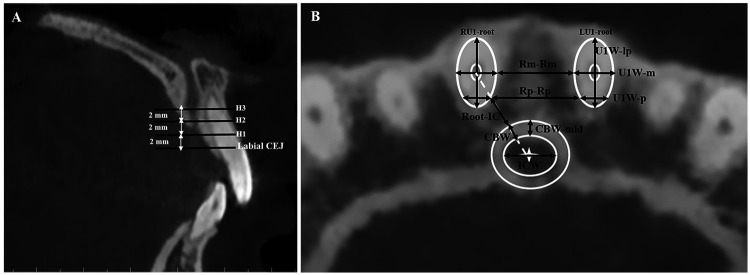
Linear analysis of incisive canal and U1 dimensions: **(A)** levels of measurement were set at three vertical heights above the labial cementoenamel junction of the maxillary central incisor: H1 = 2 mm, H2 = 4 mm, and H3 = 6 mm. **(B)** Anatomical parameters measured at each level: Canal width (ICW): maximum width of the canal measured at its center along the transverse plane. Cortical bone width of the canal (CBW): thickness of the canal cortical bone at the lateral margin, measured along a line passing through the centers of both the canal and the incisor root (white arbitrary line). Root–canal sagittal distance (root-IC): shortest linear distance between the lateral boundary of the U1 root and the lateral boundary of the canal cortical bone. Cortical bone width at midsagittal plane (CBW-mid): thickness of the canal cortical bone along the midsagittal plane of the canal. U1 root widths: U1W-m, mesiodistal width at medial points; U1W-p, mesiodistal width at posterior points; U1W-lp, labiopalatal width. Inter-root distances: Rm–Rm, distance between the most medial points of the U1 roots; Rp–Rp, distance between the most posterior points of the U1 roots.

Parameters of the canal and incisor root were assessed at three axial levels above the labial cementoenamel junction of the maxillary central incisor (2 mm, 4 mm, and 6 mm; H1, H2, H3) using Invivo Dental Imaging Software (v6, Anatomage, San Jose, CA, USA) (Figure 1A). These planes align with previously validated CBCT protocols (8, 15).

At each of these levels, specific linear parameters of the canal and incisor root were measured individually to ensure clarity and reproducibility (Figure 1B). The following measurements were performed:

Incisive canal width (ICW): The maximum width of the canal was measured at its center along the transverse (horizontal) plane of the canal, regardless of the orientation of the canal lumen.
Lateral cortical bone width of the canal (CBW): The thickness of the canal cortical bone at the lateral margin, measured along a line passing through the centers of both the canal and the incisor root.
Root–canal sagittal distance: The shortest linear distance between the lateral boundary of the U1 root and the lateral boundary of the canal cortical bone, measured along a line passing through the centers of both the canal and the incisor root.
Cortical bone width at the midsagittal plane (CBW-mid): The thickness of the canal cortical bone along the midsagittal plane of the canal.
U1 root width: The width of the root was measured along the mesiodistal aspect at the medial (U1W-m) and posterior (U1W-p) points, and along the labiopalatal aspect (U1W-lp).
Inter-root distances: Distances between the most medial (Rm–Rm) and posterior (Rp–Rp) points of the U1 roots.

The length of the U1 root was measured from the crown tip to the root tip along the tooth's long axis in the sagittal plane. Canal height was defined as the vertical distance between the incisive foramen plane and the palatal cementoenamel junction (PCEJ).

**Figure 2:**
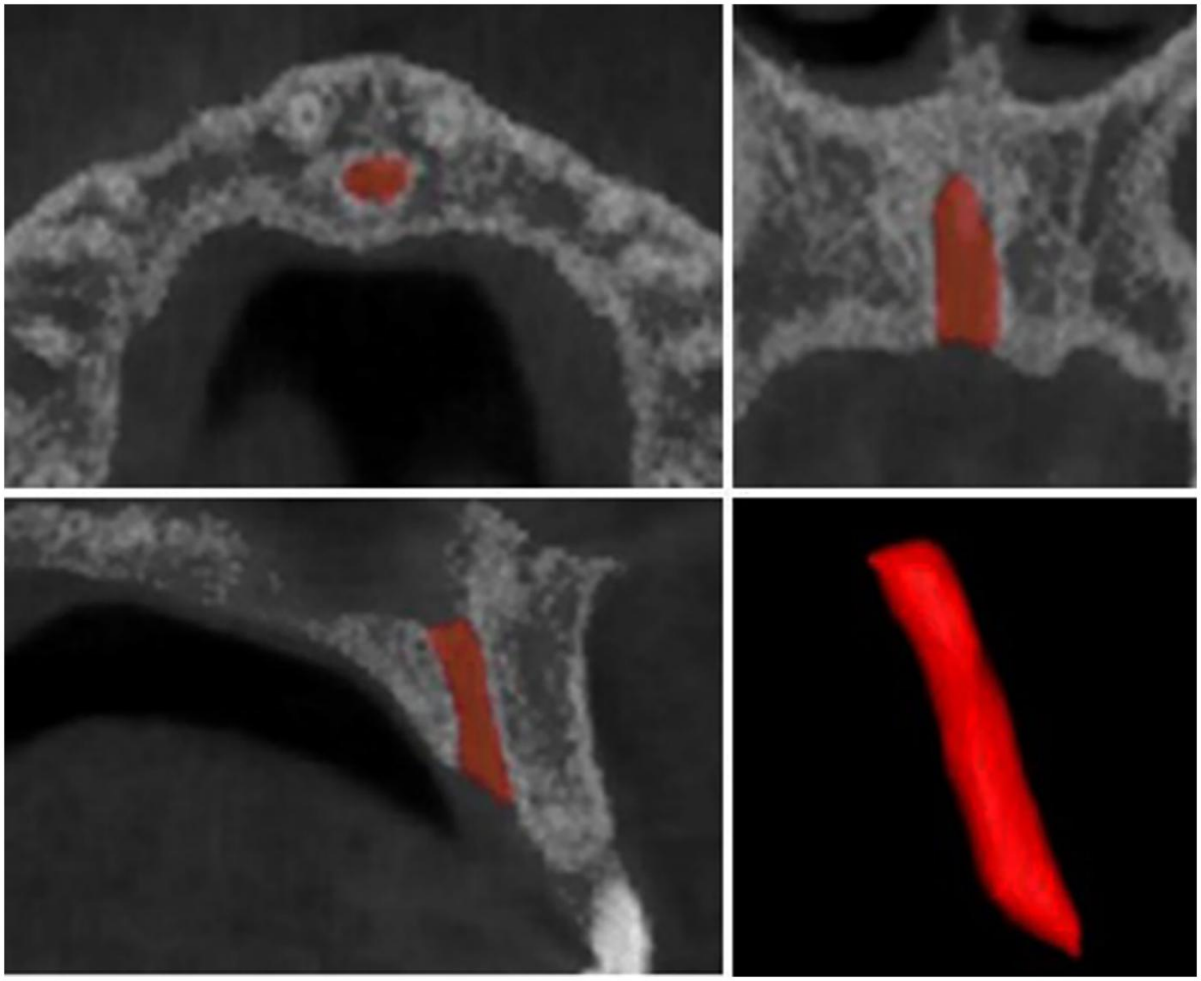
Three-dimensional segmentation of the internal portion of the incisive canal and the reconstructed model used for volumetric analysis.

**Figure 3:**
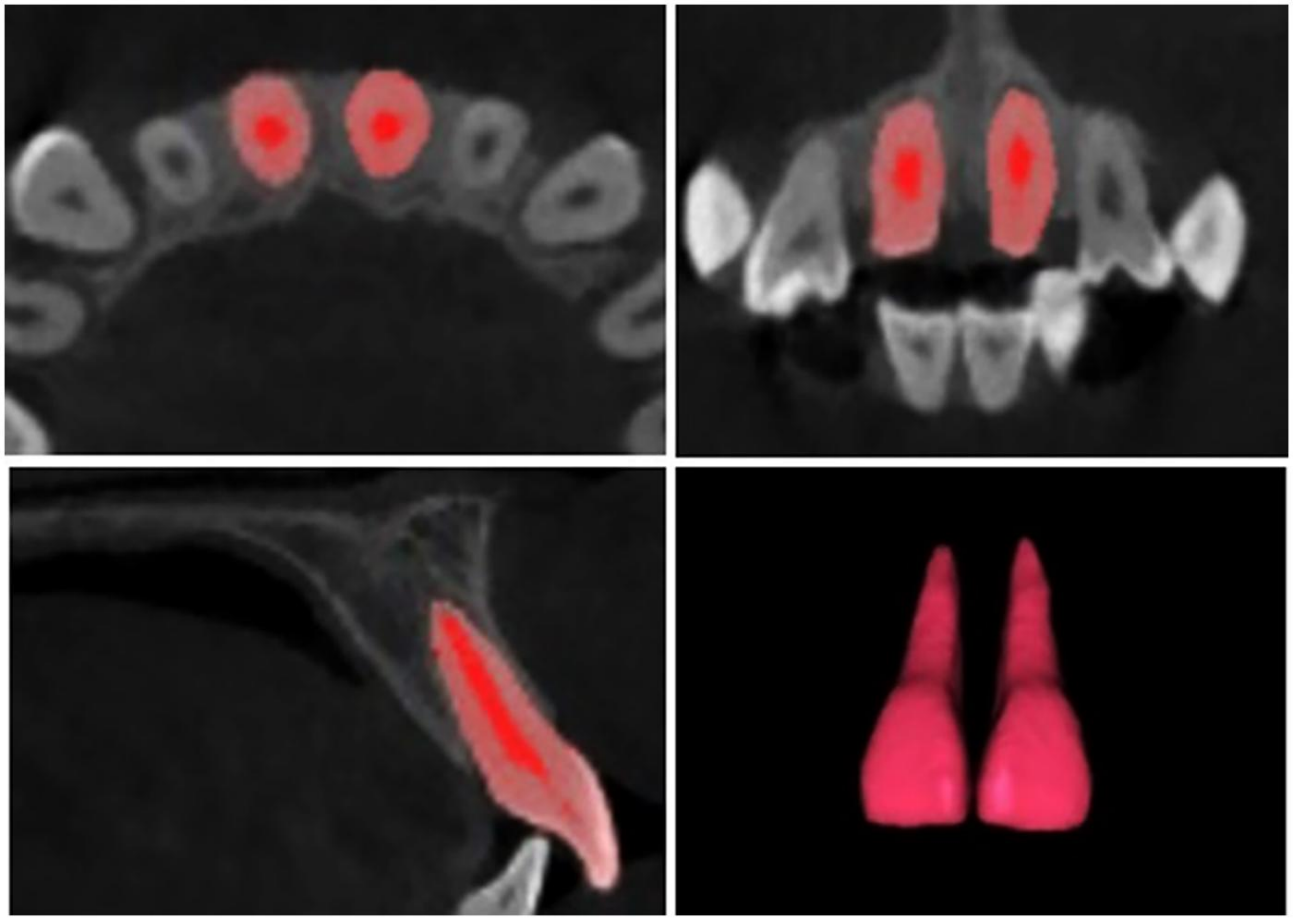
Three-dimensional segmentation of the maxillary central incisors and the reconstructed model applied for volumetric analysis.

Volumetric assessments of the IC and U1 were conducted using three-dimensional segmentation and image analysis software (Mimics 21, Materialise, Leuven, Belgium). The IC was delineated from the nasal cavity floor superiorly to the palatal roof inferiorly (Figure 2), while the U1 was segmented from the incisal edge to the root apex (Figure 3). Based on these segmentations, 3D volumetric models of both structures were reconstructed, allowing automated computation of their respective volumes and surface areas.

**Figure 4:**
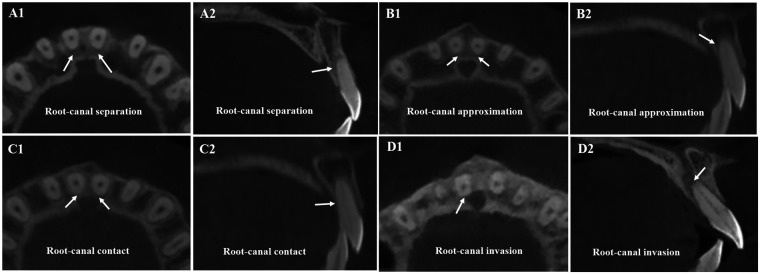
Examples of different root-canal proximity patterns: (**A**1, **A**2) root-canal separation (arrows). (**B**1, **B**2) Root-Canal approximation (arrows). (**C**1, **C**2) Root-Canal contact (arrows). (**D**1, **D**2) Central incisor root invasion into the canal (arrows).

Root–canal proximity patterns were classified as separation, approximation, contact, or invasion based on changes in root–canal distance between pre- and post-treatment CBCT (Figure 4). A root-canal distance of 0 mm indicated contact. Negative values indicated invasion, with CBW recorded as zero if the canal lumen was reached. Positive values indicated separation.

**Figure 5:**
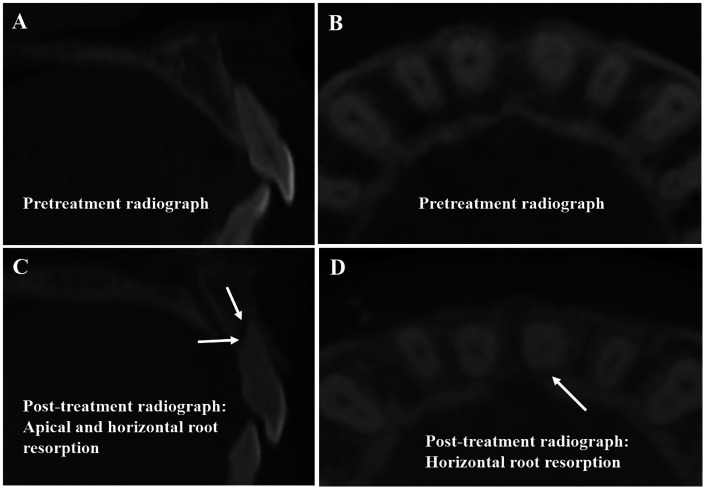
Examples of U1 root resorption: **(A,B)** pretreatment radiographs. **(C,D)** Post-treatment radiographs showing apical and horizontal root resorption (arrows).

Root resorption was assessed separately in terms of root length and root width using CBCT measurements (Figure 5). Changes in root length, corresponding to apical root resorption, were considered the primary indicator of root resorption, as changes in root width (Horizontal root resorption) were minimal and not statistically significant between groups. Additional data included amount of incisor movement along the Frankfort plane based on T1-T2 CBCT superimpositions, SNA angle, U1–SN angle, and treatment duration.

### Statistical analysis

SPSS v25.0 was used. Shapiro–Wilk test showed non-normal distribution; thus, non-parametric tests were applied. Mann–Whitney U and Wilcoxon signed-rank tests compared variables between/within groups. Chi-square tested categorical outcomes (e.g., root-canal proximity patterns). Kruskal–Wallis with Tukey's *post hoc* test compared resorption across root-canal categories. Logistic regression identified predictors of root resorption and negative U1–canal distance (i.e., proximity, contact, or invasion). The significance threshold was set at *P* < 0.05. To mitigate over-testing, Bonferroni correction was applied.

The final sample of 100 patients exceeded the minimum requirement calculated by G*Power (26 subjects per group; *α* = 0.05, power = 95%), ensuring sufficient power and adequate events per variable for multivariate regression. Predictors entered into the regression model were selected if they demonstrated significant associations in univariate analysis or had strong clinical relevance based on previous literature and biological plausibility. These included demographic factors (age), treatment-related variables (duration, U1 movement), skeletal parameters (SNA, U1-SN), dental parameters (root length, canal dimensions, U1–canal distance), and occlusal characteristics (ABO index score).

All tests were two-tailed. Two calibrated examiners (R.K.A. and K.A.S.) performed all measurements. Both examiners completed an 8-hour training session using 20 practice CBCT scans. Intra-examiner repeatability was assessed on 15% of the cases after a 2-week interval. Inter- and intra-examiner reliability were evaluated using intraclass correlation coefficients (ICC), which demonstrated excellent agreement, with all ICC values exceeding 0.86.

## Results

### Descriptive data

The final sample included 100 patients (200 central incisors), divided into a non-extraction group (*n* = 60) and an extraction group (*n* = 40). Baseline demographic characteristics and ABO discrepancy index scores were almost comparable between groups (*P* > 0.05), except for treatment duration, anterior tooth movement, anterior open bite, and vertical skeletal pattern (SN-GoGn angle) (all *P* < 0.05). The average amount of maxillary incisor retraction was 2.44 ± 1.68 mm in the extraction group and 1.19 ± 0.95 mm in the non-extraction group. Detailed descriptive statistics are presented in Table 1.

**Table 1:** Participant demographics and initial ABO discrepancy index scores of the two groups.

Item	Non-extraction (*n* = 60)	Extraction (*n* = 40)	*P* value
Male	18 (30)	15 (37.5)	0.516
Female	42 (70)	25 (62.5)	
Age, y	22.73 ± 7.48	22.54 ± 7.25	0.764
Treatment duration, y	2.02 ± 7.90	2.93 ± 1.35	0.001*
U1 move, mm	−1.19 ± 0.95	−2.44 ± 1.68	<0.001*
ABO score			
Overjet	1.37 ± 1.09	1.68 ± 1.51	0.314
Overbite	1.50 ± 1.13	1.65 ± 1.51	0.937
Anterior open bite	0.00 ± 0.00	0.75 ± 1.97	0.003*
Posterior open bite	0.00 ± 0.00	0.00 ± 0.00	1.000
Crowding	0.53 ± 0.85	1.00 ± 0.78	0.114
Occlusion	4.07 ± 1.33	4.20 ± 2.11	0.889
Posterior crossbite	0.00 ± 0.00	0.00 ± 0.00	1.000
ANB	2.53 ± 2.65	2.42 ± 2.66	0.805
SN-GoGn	1.75 ± 2.48	0.46 ± 0.89	0.012*
IMPA	3.12 ± 3.66	2.65 ± 3.40	0.439
Total ABO score	14.86 ± 6.40	15.57 ± 6.14	0.605

All variables are reported as mean ± SD but gender as frequency (rate.). *P* value: Chi-square test for gender, Mann–Whitney *U*-test for all other variables.

* Significant *P*-values.

### Changes in IC and U1 dimensions

**Figure 6:**
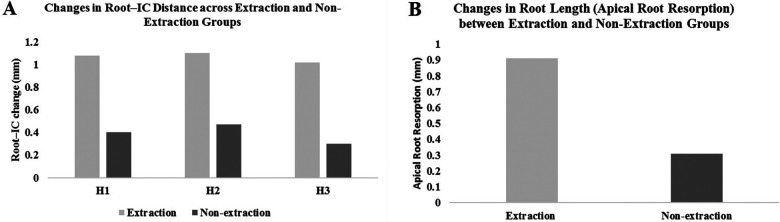
Bar charts showing comparison of root–canal distance changes and apical root resorption between extraction and non-extraction groups: **(A)** changes in root–canal distance across categories H1, H2, and H3, with extraction cases demonstrating consistently greater reductions compared with non-extraction cases. **(B)** Mean changes in root length (apical root resorption), with extraction cases showing significantly greater resorption compared with non-extraction cases.

As shown in Table 2, canal width generally increased in the non-extraction group but significantly decreased in the extraction group, particularly at H1 (*P* *<* 0.05). CBW showed a significant reduction at all levels in the extraction group (*P* < 0.05), and at H1 in the non-extraction group (*P* < 0.05). The root–canal distance decreased significantly in both groups, with greater reductions in the extraction group at H1 (1.08 ± 0.92 mm vs. 0.40 ± 0.58 mm, *P* < 0.05), H2 (1.10 ± 0.91 mm vs. 0.47 ± 0.78 mm, *P* < 0.05), and H3 (1.02 ± 0.99 mm vs. 0.30 ± 0.98 mm, *P* < 0.05) (Figure 6A). Apical root resorption, reflected by root length reduction, declined post-treatment, with significantly greater reduction in the extraction group (0.91 ± 0.80 mm) than in the non-extraction group (0.32 ± 0.56 mm) (*P* <  0.05) (Figure 6B). Importantly, these root and canal changes were predominantly observed in cases undergoing larger magnitudes of incisor retraction supported by miniscrew anchorage. In the extraction group, the subgroup exhibiting the greatest changes, characterized by an average of 3.46 ± 1.66 mm of retraction, accounted for most instances of canal contact/invasion.

**Table 2:** Comparison of changes in incisive canal and U1 dimensions between extraction and non-extraction groups.

Measure, mm	Non-extraction			Extraction			*P*-value^b^
	T1	T2	*P*-value^a^	T1	T2	*P*-value^a^	
Canal width							
H1	3.81 ± 0.68	3.95 ± 0.75	0.056	3.87 ± 0.82	3.49 ± 0.62	<0.001*	<0.001*
H2	3.23 ± 0.79	3.31 ± 0.76	0.076	3.49 ± 0.71	3.42 ± 0.72	0.312	0.027*
H3	3.02 ± 0.88	3.07 ± 0.79	0.247	3.20 ± 0.67	3.13 ± 0.64	0.296	0.089
Canal cortical bone width (CBW)							
H1	0.96 ± 0.20	0.95 ± 0.20	0.822	1.01 ± 0.20	0.59 ± 0.42	<0.001*	<0.001*
H2	1.09 ± 0.22	1.02 ± 0.24	0.046*	0.98 ± 0.19	0.69 ± 0.38	<0.001*	<0.001*
H3	0.97 ± 0.24	1.06 ± 0.26	0.015*	0.95 ± 0.21	0.77 ± 0.28	<0.001*	<0.001*
Canal cortical bone width-mid (CBW-Mid)							
H1	0.99 ± 0.23	1.03 ± 0.22	0.111	1.00 ± 0.24	0.78 ± 0.24	<0.001*	<0.001*
H2	1.16 ± 0.27	1.05 ± 0.30	<0.001*	0.98 ± 0.21	0.79 ± 0.24	<0.001*	0.310
H3	1.08 ± 0.23	1.11 ± 0.33	0.662	0.95 ± 0.20	0.90 ± 0.18	0.359	0.296
U1 root-canal distance							
H1	1.54 ± 0.72	1.14 ± 0.61	<0.001*	1.40 ± 0.83	0.32 ± 0.67	<0.001*	<0.001*
H2	2.02 ± 1.00	1.55 ± 1.00	<0.001*	1.94 ± 1.01	0.84 ± 1.00	<0.001*	<0.001*
H3	2.54 ± 1.24	2.24 ± 1.20	0.087	2.61 ± 1.22	1.59 ± 1.32	<0.001*	0.001*
Root length	21.53 ± 1.56	21.22 ± 1.60	<0.001*	21.51 ± 2.06	20.60 ± 2.32	<0.001*	<0.001*
Root width-							
H1	4.08 ± 0.42	3.95 ± 0.37	0.002*	4.00 ± 0.52	3.78 ± 0.47	0.001*	0.112
H2	3.22 ± 0.45	3.24 ± 0.43	0.393	3.06 ± 0.50	3.04 ± 0.47	0.752	0.670
H3	2.32 ± 0.52	2.26 ± 0.51	0.596	2.30 ± 0.42	2.17 ± 0.82	0.960	0.348

* Significant *P*-values.

a Wilcoxon ranks paired test.

b Mann–Whitney *U*-test comparing changes (T2-T1) between the two group.

Both treatment groups exhibited reductions in IC and U1 volumes and surface areas after treatment, with more pronounced changes in the extraction group. The IC area significantly decreased in the non-extraction group (*P* = 0.015). The U1 volume decreased significantly in both groups (*P* = 0.022 non-extraction; *P* = 0.045 extraction), whereas U1 surface area showed a significant decline only in the extraction group (*P* < 0.001). Between-group comparisons confirmed greater overall volumetric and surface area reductions in the extraction group (all *P* < 0.05), indicating enhanced morphological remodeling following extraction-based retraction (Table 3).

**Table 3:** Comparison of incisive canal and central incisor volumes (mm³) and surface areas (mm^2^) at T1 and T2 between treatment groups.

Parameter	Non-extraction			Extraction			*P*-value^b^
	T1	T2	*P*-value^a^	T1	T2	*P*-value^a^	
IC volume	113.89 ± 51.84	109.16 ± 59.91	0.056	126.31 ± 38.62	117.40 ± 32.48	0.091	0.006*
IC area	149.14 ± 38.81	143.71 ± 47.95	**0.015** *	155.28 ± 30.38	148.45 ± 25.86	0.108	0.015*
U1 volume	523.70 ± 68.26	520.32 ± 70.26	**0** **.** **022** *	534.82 ± 70.40	512.95 ± 90.96	0.045*	<0.001*
U1 area	420.81 ± 44.67	420.10 ± 41.14	0.528	427.80 ± 40.63	420.55 ± 61.77	<0.001*	<0.001*

* Significant *P*-values.

a Wilcoxon ranks paired test comparing T1 vs T2 within group.

b Mann-Whitney *U*-test comparing changes (T2-T1) between the two groups.

### Root-Canal proximity patterns

The rates of post-treatment root-canal proximity patterns among the 200 central incisors analyzed were 26.5% separation, 65.8% approximation, 4% contact, and 3.7% invasion (Table 4). Root-canal proximity patterns were significantly different between groups (*P* < 0.05), with higher contact and invasion rates in the extraction group. After Bonferroni correction for multiple comparisons across the three levels (adjusted *α* = 0.0167), the differences across all levels remained statistically significant. These close proximity patterns were concentrated among cases with greater retraction amounts, again indicating that proximity is movement-dependent rather than an inherent feature of extraction-based aligner therapy.

**Table 4:** Post-treatment comparison of root–canal proximity patterns between the studied groups across H1, H2, and H3 levels.

Root-canal proximity pattern	Non-extraction (*n* = 120 centrals)	Extraction (*n* = 80 centrals)	*P*-value
H1			<0.001*
Separation	28 (23.3)	10 (12.5)	
Approximation	92 (76.7)	40 (50)	
Contact	0 (0)	18 (22.5)	
Invasion	0 (0)	12 (15)	
H2			<0.001*
Separation	44 (36.7)	10 (12.5)	
Approximation	74 (61.7)	58 (72.5)	
Contact	2 (1.7)	2 (2.5)	
Invasion	0 (0)	10 (12.5)	
H3			<0.001*
Separation	59 (49.2)	8 (10)	
Approximation	61 (50.8)	70 (87.5)	
Contact	0 (0)	2 (2.5)	
Invasion	0 (0)	0 (0)	

Root-Canal proximity pattern is reported by frequency (rate); *P*-values: by Chi-square tests.

* Significant *P*-values.

### Apical root resorption by proximity pattern

Significant apical root resorption was observed across different root-canal proximity patterns at all levels (*P* < 0.05) (Table 5). Following Bonferroni correction (*α* = 0.0167), only the H1 and H2 level comparisons remained significant, while H3 lost significance. Tukey *post hoc* tests confirmed that invasion cases had significantly greater resorption than separation or approximation groups (*P* < 0.05). Notably, the highest resorption (1.43 ± 0.45 mm) occurred in cases with canal invasion at the H2 level.

**Table 5:** Comparison of apical root resorption across different root-canal proximity patterns at H1, H2, and H3 levels.

Root-canal proximity pattern	U1 root resorption, mm	*P*-value
H1		<0.001*
Separation (*n* = 38)	0.39 ± 0.63^a^	
Approximation (*n* = 132)	0.54 ± 0.54^ab^	
Contact (*n* = 18)	1.01 ± 0.87^bc^	
Invasion (*n* = 12)	1.38 ± 0.94^c^	
H2		0.010*
Separation (*n* = 54)	0.47 ± 0.70^a^	
Approximation (*n* = 132)	0.56 ± 0.73^a^	
Contact (*n* = 4)	1.04 ± 0.84^ab^	
Invasion (*n* = 10)	1.43 ± 0.45^b^	
H3		0.031*
Separation (*n* = 67)	0.38 ± 0.40^ab^	
Approximation (*n* = 131)	0.63 ± 0.84^b^	
Contact (*n* = 2)	0.03 ± 0.00^a^	
Invasion (*n* = 0)		

Root resorption is reported as mean ± SD; *P*-values calculated by Kruskal–Wallis test, followed by Tukey's *post hoc* test.

abc Represents the results of *post hoc* tests (Tukey).

* Significant *P*-values.

### Factors influencing U1–canal proximity and apical root resorption

Univariate analysis indicated factors such as age, treatment duration, U1 tooth movement, U1 length, canal height, canal width, U1-canal distance, ABO index score, and SNA angle were significantly associated with a negative U1-canal distance (proximity, contact, or invasion, *P* < 0.05) (Table 6). However, after Bonferroni correction (*α* = 0.004), treatment duration, U1 movement, U1 root length, canal width, U1-canal distance, ABO index score, and SNA angle remained significant (*P* < 0.004). Multivariate logistic regression identified the most influential factors associated with a negative U1-canal distance (*P* < 0.05) were treatment duration (OR = 0.662, 95% CI = 0.456–0.961), U1 tooth movement (OR = 43.216, 95% CI = 17.632–105.921), canal height (OR = 0.475, 95% CI = 0.329–0.687), U1-canal distance (OR = 3.807, 95% CI = 2.131–6.800), ABO index score (OR = 1.124, 95% CI = 1.039–1.215), and SNA angle (OR = 0.880, 95% CI = 0.774–0.999). After adjusting for multiple testing (*α* = 0.006), U1 movement, canal height, U1-canal distance, and ABO index score remained significant.

**Table 6:** Univariate and multivariate logistic regression analysis identifying factors associated with negative U1–canal interactions.

Factor	Root proximity/contact/ invasion		Univariate analysis	Multivariate analysis		
	No (159)	Yes (441)	*P*-value	*P*-value	OR	95% CI
Age, y	22.23 ± 7.59	24.28 ± 7.86	0.005*	0.096	0.951	0.897–1.009
Treatment duration, y	2.08 ± 0.75	2.50 ± 1.18	<0.001*	0.030*	0.662	0.456–0.961
U1 movement, mm	−0.31 ± 0.78	−2.06 ± 1.48	<0.001*	<0.001*	43.216	17.632–105.921
U1 root length, mm	20.72 ± 2.09	21.88 ± 1.61	<0.001*	0.535	0.939	0.768–1.147
Rm-Rm distance, mm	3.59 ± 0.94	3.70 ± 0.93	0.204			
Rp-Rp distance, mm	4.26 ± 0.80	4.35 ± 0.89	0.320			
Canal height, mm	4.22 ± 1.17	4.52 ± 1.35	0.014*	<0.001*	0.475	0.329–0.687
Canal width, mm	3.60 ± 0.69	3.35 ± 0.63	<0.001*	0.106	0.574	0.293–1.125
U1-Canal distance, mm	1.80 ± 0.82	2.23 ± 0.95	<0.001*	<0.001*	3.807	2.131–6.800
ABO index score	13.07 ± 6.88	15.89 ± 5.84	<0.001*	0.003*	1.124	1.039–1.215
SNA ( °)	81.48 ± 4.28	83.66 ± 3.82	<0.001*	0.049*	0.880	0.774–0.999
U1-SN ( °)	102.88 ± 7.43	101.84 ± 9.45	0.207			

OR, Odds ratio; CI, Confidence interval. All factors were reported as mean ± SD.

* Significant *P*-values (<0.05).

Regarding apical root resorption, age, treatment duration, U1 movement, U1 root length, canal width, and U1-SN angle showed positive associations with root resorption as revealed by the multivariate linear regression analysis (*P* < 0.05) (Table 7). After adjusting for multiple testing (*α* = 0.007), treatment duration, U1 movement, U1 root length, canal width remained significant, underscoring the biomechanical and anatomical drivers of root resorption. This indicates that larger retraction distances and longer movement paths, rather than therapy itself, were primary contributors to increased root resorption risk.

**Table 7:** Multiple linear regression analysis identifying potential factors contributing to apical root resorption.

Factor	Initial model			Final model		
	Beta	*P*-value	95% CI	Beta	*P*-value	95% CI
Age	0.009	0.027*	0.001–0.016	0.009	0.010*	0.002–0.016
Treatment duration, y	0.190	<0.001*	0.141–0.238	0.176	<0.001*	0.132–0.221
U1 movement, mm	0.155	<0.001*	0.112–0.198	0.150	<0.001*	0.117–0.183
U1 root length, mm	0.070	<0.001*	0.041–0.099	0.061	<0.001*	0.034–0.088
Canal height, mm	−0.035	0.098	−.076–0.006			
ABO index score	−0.025	<0.001*	−.035–.015	−.024	<0.001*	−.033–.016
SNA ( °)	0.001	0.836	−.012–0.015			
U1-SN ( °)	0.005	0.060	0.000–0.011	0.006	0.016*	.001–0.012
Canal width, mm	0.155	<0.001*	0.081–0.228	0.130	<0.001*	.060–0.201
U1-Canal distance, mm	−0.061	0.083	−.130–0.008			
Rm-Rm distance, mm	−0.053	0.606	−.253–0.148			
Rp-Rp distance, mm	0.076	0.485	−.138–0.290			

CI, Confidence interval. All factors were reported as mean ± SD.

* Significant *P*-values (<0.05).

## Discussion

This study provides a comprehensive assessment of maxillary incisor retraction using clear aligners, highlighting both anatomical and clinical outcomes, particularly in relation to the incisive canal and apical root resorption. Consistent with previous reports using fixed appliances (6, 8, 12), extraction cases in our study exhibited decreased root–canal distances, greater cortical bone remodeling, and more frequent root–canal contact or invasion compared to non-extraction cases. From a bone physiology standpoint, these findings demonstrate the adaptive response of the anterior maxillary cortex to orthodontic loading, where localized resorption and apposition occur as a coupled remodeling process to accommodate tooth movement within biological limits. It is important to note that the more pronounced canal approximation and remodeling observed in this study were predominantly associated with cases requiring large retraction supported by TAD anchorage, rather than typical aligner cases with mild-to-moderate anterior movement. This aligns with randomized trials showing that skeletal anchorage systems producing intrusive force vectors result in higher volumetric root resorption of maxillary incisors during en-masse retraction (10).

Significant reductions in canal width and cortical bone width were observed in both groups, with more pronounced changes in the extraction group. Similar patterns were reported by Chung et al. (9), who demonstrated that maximum anterior retraction induces canal remodeling and cortical thinning, potentially increasing apical root stress. These morphological alterations reflect the mechanobiological remodeling of the maxillary alveolar and incisive canal walls under tensile and compressive strain, illustrating that the canal is not a static structure but participates dynamically in load redistribution during tooth retraction. Our findings extend these observations to clear aligner therapy, showing that although anatomical adaptations occur, the magnitude of changes is generally lower than those observed in fixed appliance studies. This likely reflects differences in force application: clear aligners deliver intermittent, lighter forces through plastic flexure and attachment engagement, whereas fixed appliances exert continuous archwire-mediated forces, which may accelerate bone remodeling and root resorption. These differences are consistent with findings that frictionless mechanics or T-loops generate greater anchorage loss and molar rotation but similar root resorption when compared with friction mechanics, emphasizing that force system design modifies load transmission to the anterior maxilla (16).

Root–canal distance decreased significantly in both groups, with intergroup differences following a consistent pattern of greater approximation in extraction cases (e.g., 1.08 mm vs. 0.40 mm at H1; 1.10 mm vs. 0.47 mm at H2; 1.02 mm vs. 0.30 mm at H3). While these differences are submillimeter and approach CBCT voxel resolution, they remain clinically relevant because previous studies indicate that root proximity under 1 mm increases the risk of contact or invasion (6–8). Finite-element evidence suggests that clear aligner retraction under strong anchorage still allows some mesial movement of posterior teeth due to reciprocal forces, contributing to anterior root approximation in challenging extraction cases (11). These anatomical changes should also be interpreted in the context of the mean amount of maxillary incisor retraction, which was substantially higher in extraction cases (2.44 ± 1.68 mm) than in non-extraction cases (1.19 ± 0.95 mm), with the subgroup exhibiting contact or invasion showing the greatest movement (3.46 ± 1.66 mm).

Contact and invasion patterns were significantly more frequent in the extraction group, aligning with previous reports in fixed appliance therapy (8). Further, the observed contact and invasion events should not be generalized to all aligner treatments but rather to cases involving substantial anterior retraction demands combined with TAD-assisted bodily movement mechanics. Notably, the incidence of root–canal contact in our clear aligner-treated extraction cases (12.5%) was substantially lower than in fixed appliance reports (44.3% in Yu et al., 2022) (8), while invasion rates were slightly higher (9.1% vs. 6.4%). These patterns suggest that even under physiologic force magnitudes, bone remodeling near the canal is spatially constrained, and its remodeling capacity may vary according to local cortical density and vascularization. This is further supported by palatal anchorage device-assisted aligner studies showing that posterior anchorage reinforcement changes stress concentration patterns on the incisors, potentially influencing apical load distribution near the canal (17).

Apical root resorption was significantly higher in extraction cases (0.91 ± 0.80 mm vs. 0.32 ± 0.56 mm in non-extraction cases). These values are notably lower than those reported in fixed appliance studies (Yu et al., 2022: 2.3 ± 1.4 mm for extraction, 1.1 ± 0.75 mm for non-extraction), likely due to the intermittent nature of aligner forces compared with the continuous forces of fixed appliances. These findings align with many other studies (18–21). Among them, a recent systematic review and meta-analysis by Butsabul et al. (18), reported that clear aligner therapy causes minimal apical root resorption, with the maxillary central incisors showing the greatest reduction in root length (mean difference = 0.56 mm). Together, these findings support the notion that appliance type modulates the severity of root resorption, even when similar retraction magnitudes are applied. Furthermore, the pattern of increased resorption with extraction and greater movement mirrors trends reported by Al-Rokhami et al. (12, 20), reinforcing the role of biomechanical and anatomical factors in predicting resorption risk.

Importantly, the volumetric assessment of both the incisive canal and the maxillary incisor roots provides a physiologically relevant perspective on bone and tooth tissue remodeling. The present study revealed significant post-treatment reductions in canal volume and surface area, particularly in extraction cases, indicating adaptive cortical bone resorption accompanied by compensatory appositional activity at adjacent regions. Conversely, a modest reduction in incisor root volume and surface area was observed, consistent with localized apical resorption. These 3D volumetric patterns illustrate how the bone–tooth complex undergoes coordinated morphological adaptation under orthodontic loading, integrating osteoclastic activity near pressure zones and osteoblastic remodeling on tension sides. Volumetric root loss patterns similar to ours have been reported in skeletal-anchored retraction systems, where centrals and canines show the greatest 3D root loss under intrusive or oblique force vectors (10), supporting our findings that anatomical constraints guide remodeling.

Multivariate analysis identified treatment duration, U1 movement (amount of retraction), initial root length, and canal width as significant predictors of apical root resorption (*P* <  0.05), even after correcting for multiple testing (*α* = 0.007). Notably, greater incisor movement (*β* = 0.150) and longer treatment duration (*β* = 0.176) were the strongest predictors. This confirms the dose–response nature of bone remodeling: prolonged loading and greater displacement intensify localized strain, triggering remodeling cascades that extend from the periodontal ligament to the adjacent cortical boundary. Canal width similarly influenced resorption risk, suggesting that anatomical variation dictates how applied forces distribute across root and surrounding bone.

Although U1–canal distance and canal height were not statistically significant in the final model, their borderline associations (*P* = 0.083 and *P* = 0.098, respectively) suggest indirect roles in cortical bone resistance or invasion risk. Smaller canal height was associated with increased risk of root–canal proximity (OR = 0.475, *P* < 0.05), consistent with Pan et al. (6), who reported that lower-positioned incisive foramina increase the likelihood of canal contact during retraction. The volumetric and morphometric findings together emphasize that individual bone physiology, especially local cortical thickness, canal orientation, and bone density, critically determines the biological limits of safe orthodontic tooth movement.

Overall, our results indicate that clear aligner therapy, while producing similar directional changes as fixed appliances, is associated with lower root–canal contact rates and reduced apical resorption. When integrated with volumetric evidence, these outcomes highlight that clear aligner forces evoke a more balanced bone turnover, minimizing excessive osteoclastic activity while allowing sufficient remodeling for tooth translation. Clinically, these findings underscore the importance of assessing canal morphology, root proximity, and regional bone physiology during treatment planning to minimize the risk of root resorption and canal invasion, in alignment with recent recommendations for biologically guided orthodontic approaches (18, 22). These findings collectively reinforce that incisive canal approximation is a function of movement magnitude and anatomical constraints, not of therapeutic appliance itself, and becomes clinically relevant mainly when large bodily retraction is planned with adjunctive anchorage. Systematic reviews reinforce this principle, demonstrating that TSADs significantly improve torque preservation and anchorage but do not eliminate biomechanical limits imposed by cortical anatomy (23).

This study's retrospective design limited access to certain clinical variables, including pulp vitality, periodontal condition, and occlusal forces, which may also influence resorption risk. In addition, treatment-related variables such as the magnitude of applied forces and patient compliance with aligner wear were not controlled. Although volumetric analysis provided a more physiologic representation of bone remodeling, segmentation and voxel resolution constraints remain potential sources of measurement bias. Future studies should integrate dynamic bone modeling, histomorphometric validation, and follow-up imaging to clarify how orthodontic forces regulate bone metabolism and canal adaptation *in vivo*.

## Conclusions

Compared to non-extraction protocols, maxillary central incisor retraction in extraction cases using clear aligners with miniscrew-assisted anchorage produced greater reductions in root–canal distance and canal cortical bone width, accompanied by higher levels of apical root resorption. These changes were most pronounced in cases requiring larger retraction distances, consistent with the movement-dependent nature of bone remodeling and root-canal proximity. Complementary volumetric analysis demonstrated coordinated decreases in incisive canal and incisor-root volumes, reflecting physiologic remodeling within biologically acceptable limits. Treatment duration, the magnitude of incisor movement, initial canal height and width, root length, and pre-treatment root–canal distance were significant predictors of both apical root resorption and canal proximity, underscoring the central role of both biomechanics and anatomy in governing tissue response during clear-aligner-based retraction.

## Data Availability

The original contributions presented in the study are included in the article/Supplementary Material, further inquiries can be directed to the corresponding author.

## Supplementary material

Supplementary Figure 1 Clinical photographs illustrating the miniscrew-assisted retraction mechanics used with clear aligners.
