# Supplementary figures and images for: Three-dimensional CBCT analysis of bone remodeling, root resorption, and incisive canal morphology during miniscrew-assisted aligner-based incisor retraction with and without extractions
Source: Front Oral Health. 2026 Feb 2;7:1728205. doi: 10.3389/froh.2026.1728205 (PMC12907424; doi:10.3389/froh.2026.1728205)

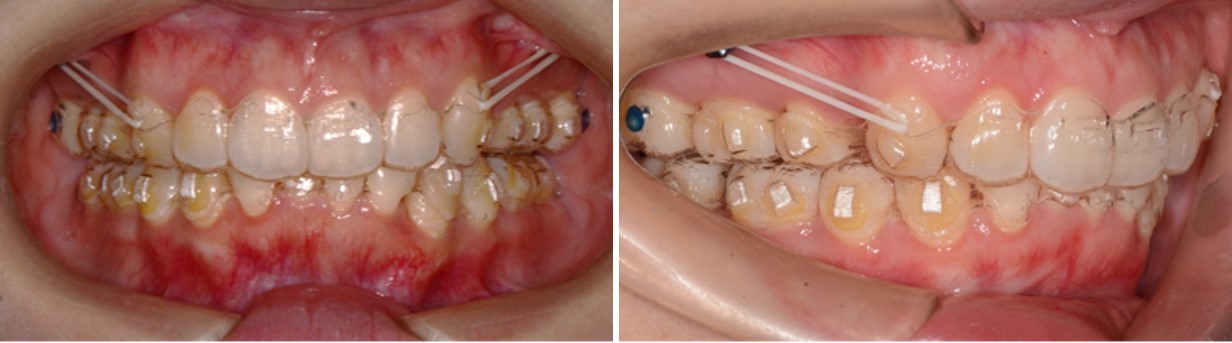

Supplement: Supplementary Figure 1 — Clinical photographs illustrating the miniscrew-assisted retraction mechanics used with clear aligners. [file Image1.jpeg]
